# Association of Health Information Literacy and Health Outcomes Among Individuals with Type 2 Diabetes and Metabolic Syndrome

**DOI:** 10.3390/nursrep15030090

**Published:** 2025-03-05

**Authors:** Kailu Wu, Xiaoyan Qi, Aihua Li, Huan Dong, Xiaojing Wang, Meihua Ji

**Affiliations:** 1 School of Nursing, Capital Medical University, 10 You-An-Men Wai Xi-Tou-Tiao, Feng-Tai District, Beijing 100069, China; 15611757694@163.com (K.W.); qxyan0626@163.com (X.Q.); 2 Center for Endocrine Metabolism and Immune Diseases, Beijing Luhe Hospital, Capital Medical University, 82 Xin-Hua-Nan-Lu, Tongzhou District, Beijing 101199, China; aihuali2025@163.com (A.L.); dhwf91@163.com (H.D.)

**Keywords:** nursing, type 2 diabetes, metabolic syndrome, health information literacy, glycemic control

## Abstract

**Objectives**: Based on social cognitive theory, this study aims to explore the associated factors of and whether and how health information literacy was correlated to health behavior and glycemic control among individuals with type 2 diabetes and metabolic syndrome. **Methods**: Following convenient sampling, this cross-sectional, correlational study was conducted among 225 patients with type 2 diabetes and metabolic syndrome from an outpatient clinic in a suburban area of Beijing, China. Hierarchical multiple regression and mediation analysis were performed to explore the effect of health information literacy on self-management practice and hemoglobin A1c in this sample. The STROBE guidelines for cross-sectional studies were followed. **Results**: The findings showed incompetent health information literacy, inadequate self-management behavior, and suboptimal glycemic control in a sample of patients with type 2 diabetes and metabolic syndrome. Based on social cognitive theory, the results of regression analysis indicated that self-management attitude, health problem-solving, and chronic illness resources were correlated with self-management practice, and health problem-solving and health information evaluation were correlated with hemoglobin A1c. Mediation analysis revealed that self-management attitude, health problem-solving, and chronic disease resources fully mediated the effect of health information literacy on self-management practice. There was an indirect effect of health information literacy on hemoglobin A1c through health problem-solving. **Conclusions**: The findings demonstrated that health information literacy has significant indirect and direct effects on self-management behavior and glycemic control through self-management attitude, health problem-solving, and chronic disease resources in a sample of patients with type 2 diabetes and metabolic syndrome.

## 1. Introduction

Health information literacy (HIL) is essential to assist individuals in health decision-making. The concept of HIL is defined as a set of competencies in recognizing, accessing, understanding, evaluating, and applying available health information to make good health decisions to promote and maintain health [1]. While many definitions exist and evolve to accommodate the changing environment, such as eHealth literacy and digital health literacy, almost all definitions share the same core elements as HIL [2,3]. Recognized as a social determinant of health, both health literacy (HL) and HIL have been identified to influence health outcomes both directly and indirectly among different populations [4,5,6]. Limited HIL and associated adverse effects are commonly found in Chinese individuals, especially for the disadvantaged socioeconomic groups, vulnerable populations, and individuals with chronic diseases [7].

The prevalence of type 2 diabetes mellitus (T2DM) has reached about half a billion globally [8]. Metabolic Syndrome (MetS), a cluster of metabolic risk traits, including hyperglycemia, hypertension, dyslipidemia, and abdominal obesity, affects approximately 25% of the global population and more than 33.9–45.1% of Chinese population [9,10]. In Chinese individuals with T2DM, approximately 57.4–89.4% were identified to have MetS [11,12]. Individuals with T2DM and MetS have a greater risk of developing cardiovascular diseases (CVDs) due to the common underlying causal processes and synergistic effect among these conditions, compared to the general population [9]. Promoting lifestyle interventions has been recommended to improve health outcomes in individuals with MetS [13,14]. To foster healthy lifestyle modification, individuals need to acquire adequate health information and skills to participate in self-management activities [15,16].

Empirical evidence suggested that digital and visual-based interventions are effective for improving health literacy among individuals with health issues, especially in accessing and comprehension of related health information and acquiring associated technology skills [17,18]. Meanwhile, studies have shown that health literacy and related strategies have a positive impact on improving behavioral indicators and clinical parameters related to diabetes management [19,20]. However, most studies included strategies focusing on the functional health literacy, such as basic reading, writing, and numerical skills, with limited skills in interactive and critical health literacy [20,21,22]. Based on Nutbeam’s conceptual model of health literacy and related concept analysis, interactive and critical health literacy are referred as high-order competencies; they require high-order cognitive skills in appraising health information and applying them in everyday practice [23,24]. They are essential for effective disease management, particularly when patients are confronted with an overwhelming abundance of complex information on foods, medicines, and health products [25]. In contrast, individuals with higher health literacy can critically analyze information, make rational decisions, and effectively apply relevant information to address their health concerns [3]. These skills are especially important for individuals with T2DM and MetS, as multi-morbidity could further complicate their self-management process [26].

In previous research, multiple factors have been examined to explore their impact on the relationship between HIL and related health outcomes. Knowledge has been identified as an important antecedent of HIL; it is also one of the essential factors in promoting health among individuals with heart failure [27]. Self-management attitude was taken as a significant correlate of individuals’ motivation and personality traits to engage in health behaviors [28]. Health beliefs, which are closely related to attitudes, also played a significant mediating role between health-related literacy and promotion of healthy lifestyles [29]. Contextual factors, such as environmental and informational factors, as well as social support have been identified to mediate or moderate the interaction between health related literacy and health behaviors [30,31,32]. Problem-solving, a key concept in fostering effective diabetes self-management, has been qualitatively highlighted in the conceptual models and emphasized by diabetes guidelines [14,33]. In addition, health problem-solving theory has pointed out that the concept of health problem-solving is comprised of knowledge, skills, and orientation, among which the skills related to problem-solving are identified to have a significant conceptual correlation with health information literacy [34]. Previous research has demonstrated its significant relationship with glycemic control in individuals with T2DM [35]. However, the mechanism by which HIL impacts health outcomes remains to be understood, especially the high order HIL under the interactive and critical literacy domain. Meanwhile, inconsistent evidence was found on how comprehensive HIL affects health outcomes in individuals with T2DM [36,37], and there is lack of evidence in examining these variables among individuals with T2DM and MetS.

Social cognitive theory (SCT) provides a triadic reciprocal framework that explores potential personal, behavioral, and environmental factors (PBEs) that interactively operate and collectively impact on human behavioral change [38]. The SCT has been applied widely among individuals with chronic diseases [39]. For individuals with T2DM and MetS, sociodemographic factors and HIL as personal factors interplay with behavioral and environmental factors, including self-management knowledge, attitude, health problem-solving, and chronic illness resources; these PBEs may collectively influence individuals’ health outcomes, according to SCT.

Following principles of SCT and previous evidence, the primary objective of the current study was to examine whether HIL and selected study variables were correlated with health outcomes among patients with comorbid T2DM and MetS. The secondary objective was to explore separately how HIL affects self-management practice and glycemic control in this sample. We hypothesized that:

**Hypothesis** **1.** *Greater HIL would be Positively Related to Better Health Outcomes (Self-Management Practice and Glycemic Control) after controlling covariates.*

**Hypothesis** **2.** *Behavioral Factors (Self-Management Knowledge, Self-Management Attitude, Health Problem-solving) and Environmental Factors (Chronic Illness Resources) mediate the relationship between Personal Factors (Sociodemographic Factors and HIL) and Health Outcomes (Self-Management Practice and Glycemic Control) after controlling covariates.*

## 2. Materials and Methods

### 2.1. Study Design

A descriptive, cross-sectional study was conducted to examine the associations of HIL, selected study variables, and health outcomes. This study was reported following the guidelines of the Strengthening the Reporting of Observational Studies in Epidemiology (STROBE) for cross-sectional studies in Table S1 [40].

### 2.2. Setting and Sampling

A convenient sampling was used to recruit eligible participants from the Center for Endocrine Metabolism and Immune Diseases at a tertiary hospital in a suburban area of Beijing, China. Registered individuals at the center have regular follow-up visits to the clinic (at least once every three months). In this study, we used hierarchical multiple regressions to determine the potential factors related to self-management practice and glycated hemoglobin (HbA1c), therefore the sample size was estimated based on this type of statistical analysis. Using G*Power software version 3.1.9 [41], the sample size was calculated using confidence intervals to determine the effect size at 0.153 (H_1_ ρ^2^), based on the previously reported R^2^ = 0.178 according to an early study [35]. The estimated sample size should be 222 participants with an effect size of 0.3 (H_0_ ρ^2^), 14 predictors, a power of 0.8, two-tailed with alpha set at 0.05. A total of 264 eligible participants were initially enrolled in the current study with valid data from 225 participants included in the final analysis, which is satisfactory for this study.

### 2.3. Inclusion and Exclusion Criteria

Participants were included according to the following criteria: (a) a clear diagnosis of T2DM and MetS, (b) 18 years of age or older (as the prevalence of T2DM and MetS is high in this group), and (c) able to communicate in Mandarin. The diagnostic criteria of MetS refers to the National Cholesterol Education Program (NCEP) Adult Treatment Panel III (ATP-III) [9] with modified waist circumference cutoffs for the Chinese population [40]. According to the criteria, individuals with any three of the following five factors constituted a confirmed diagnosis of MetS: (a) elevated waist circumference (WC) ≥ 90 cm in men and ≥85 cm in women, (b) elevated triglycerides ≥150 mg/dL (1.69 mmol/L) or on drug treatment, (c) reduced high-density lipoprotein cholesterol (HDL) < 40 mg/dL (1.03 mmol/L) in men and <50 mg/dL (1.30 mmol/L) in women or on drug treatment, (d) elevated blood pressure (BP) ≥ 130/85 mm Hg or reported antihypertensive drug treatment, (e) elevated fasting blood glucose ≥100 mg/dL (5.6 mmol/L) or on drug treatment. In the current study, as individuals had a clear diagnosis of diabetes, those who met any two of the ATP-III criteria (a to d) were deemed eligible. Meanwhile, individuals were excluded from the study if they had hearing and/or vision impairment, severe or unstable conditions such as cerebrovascular disease, advanced liver and kidney disease (Liver dysfunction can significantly alter drug metabolism and efficacy, and the use of medications to treat these conditions can potentially confound the study results, such as on lipid profiles and other related clinical indicators), and/or mental illness; pregnant women were also excluded.

### 2.4. Instrument with Validity and Reliability

#### 2.4.1. Socio-Demographic and Clinical Information

Participants’ socio-demographic and clinical information were collected using forms developed by the investigator. The socio-demographic information included age, gender, residence type, educational level, employment status, occupation, household income, marital status, and way of medical payment.

All anthropometric measurements were taken at the outpatient clinic of the study hospital. Participants’ waist circumference (WC) was measured at the midpoint between the lower margin of the costal arch and the iliac crest. After at least five minutes of rest, individuals’ blood pressure was measured by the investigator using a calibrated electronic sphygmomanometer. Glycated hemoglobin (HbA1c) within the last three months was used to reflect individuals’ glycemic control, and HbA1c < 7% was interpreted as optimal [42]. Clinical information and laboratory results on HbA1c, high-density lipoprotein cholesterol (HDL), low density lipoprotein cholesterol (LDL), and total cholesterol and triglyceride were retrieved from the medical records by study investigators.

#### 2.4.2. Health Information Literacy

HIL was measured using the health information literacy self-rating scale (HILSS), which includes 29 items with 5 subscales: health information consciousness, health information access, health information evaluation, health information application, and health information morality [43]. The HILSS uses a five-point Likert scale ranging from 0 (completely disagree) to 1 (completely agree) with a 0.25 interval, and the total score ranges from 0 to 29, with higher scores indicating greater HIL level. Researchers have suggested that a score below 60% (17.4) is considered as incompetent in HIL [29,44]. The Cronbach’s α of the HILSS was 0.847 and was between 0.783 and 0.917 for the subscales in the original study [43]. The Cronbach’s α of the HILSS was 0.903 among Chinese individuals with T2DM [45] and was 0.844 in the current study.

#### 2.4.3. Knowledge, Attitude, and Practice

The self-management of knowledge, attitude, and practice scale for individuals with metabolic syndrome (SMKAPS-MetS) was used to collect information on individuals’ knowledge, attitude, and health behavior [46]. It comprises 46 items with 3 subscales, including knowledge, attitude, and practice. The items of subscale on knowledge are scored dichotomously (1 = correct, 0 = false) and items of both attitude and practice subscales are rated on a five-point Likert scale (0 = “strongly disagree or never”, 5 = “strongly agree or always”). The total scores of the SMKAPS-MetS range from 22 to 134, with higher scores representing greater knowledge, attitude, and better health behavior. In the current study, each of the subscales was used separately to reflect participants’ knowledge and attitude and their self-management practice in managing MetS. The Cronbach’s α of the overall scale was 0.836 in the original study [46] and 0.844 in the current study, with Cronbach’s α ranging from 0.706 to 0.815 for the subscales.

#### 2.4.4. Health Problem-Solving

The Health Problem-Solving Scale-Chinese version (HPSS-Chinese version) was applied to reflect participants’ perspectives on health problem-solving regarding health management. The original HPSS scale was developed by Hill-Briggs [47]. This scale was modified and validated among individuals with T2DM in a previous study [48], which showed acceptable reliability and validity. The HPSS-Chinese version includes 30 items with 6 subscales: rational problem-solving, positive transfer of experience/learning, avoidant problem-solving, impulsive/careless problem-solving, negative transfer of experience/learning, and negative motivation/orientation. Each item was scored on a five-point Likert scale, ranging from 0 (“not at all true of me”) to 4 (“extremely true of me”). The sum of average scores in each subscale was used to reflect participants’ health problem-solving, with a higher score indicating a greater health problem-solving. The overall Cronbach’s α of the original scale was 0.890 among individuals with diabetes [47], whereas the HPSS-Chinese version had a Cronbach’s α of 0.911 [48]; it was 0.746 in the current study.

#### 2.4.5. Chronic Illness Resources

The translated Chronic Illness Resources Survey (CIRS) was used to collect information on societal resources reflecting the environmental factors influencing the participants [49]. The Chinese version of the CIRS includes 19 items measuring 6 aspects of social resources: personal, family and friends, healthcare team, community, media policy, and community organizations [50]. All items use a 5-point Likert scale, and the total scores ranges from 19 to 95, with higher scores showing better support from the social context. Results for the overall Cronbach’s α was 0.820 in the original study [49], 0.845 in the Chinese population [50], and 0.802 in the current study.

Acceptable overall reliability was demonstrated among included instruments in the current study, and they are displayed in Table S2.

### 2.5. Data Collection

Between October 2023 and January 2024, information on study variables and health outcomes was collected using paper-pencil questionnaires and review of health records. The study investigator and clinical nurses screened prospective participants at the outpatient clinic in the study hospital. The study objectives, data collection forms, and related procedures were explained to eligible individuals before data collection. After individuals agreed to participate and signed the written informed consent, they were encouraged to complete the questionnaires face to face, which took approximately 30 to 45 min. Participants were offered to take a break during the data collection or quit if they wished. The recruitment flow chart is shown in Supplementary Figure S1.

### 2.6. Data Analysis

Epidata software version 3.1 (Epidata Association, Odense, Denmark) was used for data entry and verification of participants’ information. All statistical analysis was performed using the IBM SPSS version 26.0 (IBM Corp., Armonk, NY, USA) and Mplus Version 8.3 (Muthén and Muthén, Los Angeles, CA, USA). Among participants (*n* = 226) who completed the current study, one had missing data on HDL and the missing data of the variables were imputed by case-based mean imputation. Mahalanobis distance was used to examine outliers of this sample, with one case being excluded. Therefore, a total of 225 participants were included in the study.

Descriptive statistics were used to analyze the characteristics of the participants. The mean and standard deviation (SD), or median and interquartile range (IQR) were used to describe normally and non-normally distributed continuous variables, respectively. Frequencies and percentages were used for categorical variables. The relationship between outcome variables and sociodemographic variables were tested using the Mann–Whitney U (*Z*), Kruskal–Wallis (*H*), and Post hoc tests for categorical variables (such as gender, residence, and occupation). Variables reflecting significant findings were taken as covariates for the subsequent analyses.

Based on the SCT and previous evidence, bivariate associations between selected study variables and health outcomes (self-management practice and glycemic control) were examined using the Spearman correlation test. Using stepwise selection, hierarchical multiple regressions were used to determine the potential study variables that were significantly correlated with outcome variables after controlling covariates (Hypothesis 1). Socio-demographics and study variables with significant findings in final regression model were included in the subsequent mediation analysis.

Using path analysis, mediation models based on maximum likelihood estimation were established to explore whether behavioral and environmental factors mediated the relationship between HIL and health outcomes (self-management practice and glycemic control) after controlling sociodemographic covariates (Hypothesis 2).

The mediation effects of the independent variables on the dependent variable via the mediator were estimated by bootstrapping with 5000 bootstrap samples. If the 95% confidence intervals (CIs) of indirect effect did not include 0, they were considered statistically significant. A significance level was set as 0.05, two-tailed.

## 3. Results

### 3.1. Characteristics of the Sample

In this study, 60.9% of the participants were male, and more than half (59.6%) were aged 45 years or older. In addition, about 53.3% (*n* = 120) of the participants had education level at or above college degree. Most of the participants lived in urban areas (*n* = 166, 73.8%) and were employed full-time (*n* = 123, 54.7%). On average, participants reported the HILSS overall score of 16.83 ± 2.96, with 54.7% of participants identified with incompetent HIL.

The mean total score of self-management practice was 42.00 (IQR = 15.00) in this sample, and 37.3% of participants failed to reach the passing level of self-management behaviors in managing MetS (under 60% of the total score). The mean HbA1c was 8.80% (IQR = 3.55%), and only 22.2% reported optimal glycemic control (HbA1c < 7%). Detailed demographic and baseline clinical characteristics of study participants are shown in Table 1.

Analysis of differences among different groups showed that age, gender, employment status, and household income were found to be significantly associated with self-management practice in the current sample (all *p* < 0.05). Regarding HbA1c, statistically significant differences were found among various age groups and employment status (all *p* < 0.05). Women showed better self-management practice than men (*p* < 0.001), and those with monthly household income less than 3000 CNY had better self-management practice than those with monthly income higher than 5000 CNY. In this sample, participants older than 60 years and retired had better self-management practice and lower HbA1c than the other corresponding groups. These sociodemographic factors were treated as covariates and were included in subsequent analyses. More details can be found in Supplementary Table S3.

### 3.2. Hypothesis 1: Greater HIL Would Be Positively Related to Better Health Outcomes (Self-Management Practice and Glycemic Control) After Controlling Covariates

The results of bivariate correlational analysis among study variables are shown in Table 2. Based on these results, the overall HILSS score was positively correlated with self-management practice (r = 0.21) and negatively related to HbA1c (r = −0.15), with *p* < 0.05. Association of personal factors (r ranged from −0.21 to 0.46), behavioral factors (r ranged from 0.32 to 0.39), and environmental factors (r = 0.52) with self-management practice showed a small to moderate effect (*p* < 0.05). As for HbA1c, a slightly weaker association was identified among study variables (r from −0.35 to 0.18), *p* < 0.05, except for self-management attitude.

Following hierarchical multiple regression, using self-management behavior (the practice subscale of the SMKAPS-MetS) as the dependent variable, five factors (age, gender, years of education, employment status, and household income) were treated as covariates and introduced into Model 1 as a group. The results showed that Model 1 explained 24.9% (adjusted R^2^ = 0.23) of the variance for self-management practice. Next, the five subdomains of the HILSS, along with knowledge, attitude, health problem-solving, and chronic disease resources were added to Model 1 as a group, and the overall model improved significantly, as it explained 56.8% (adjusted R^2^ = 0.56) of the variance for self-management practice (see Model 2). These results suggested that self-management attitude, health problem-solving, and chronic disease resources were significant correlates of self-management practice in this sample (see details in Table 3). However, the individual HILSS subdomains were not significant in the final model.

Meanwhile, hierarchical multiple regression was performed to determine the correlates of HbA1c following stepwise procedure with backward deletion. In Model 1, five covariates were entered in the model, in which only age remained significant and the whole model explained 8.6% (adjusted R^2^ = 0.07) of the variance for HbA1c. In Model 2, the five HILSS subdomains, along with knowledge, attitude, health problem-solving, chronic disease resources, and self-management practice, were added to Model 1. After controlling for covariates, health information evaluation and health problem-solving were significant correlates of HbA1c (Model 2). The overall model collectively explained 16.3% (adjusted R^2^ = 0.14) of the variance for HbA1c. The results showed that for every unit increase in health information evaluation and health problem-solving, the HbA1c would decrease by 0.643 (*p* < 0.05, 95% CI: −1.161 to −0.125) and 0.119 (*p* < 0.05, 95% CI: −0.207 to −0.032), respectively. More details are shown in Table 4.

### 3.3. Hypothesis 2: Behavioral Factors (Self-Management Knowledge, Self-Management Attitude, Health Problem-Solving) and Environmental Factors (Chronic Illness Resources) Mediate the Relationship Between Personal Factors (Sociodemographic Factors and HIL) and Health Outcomes (Self-Management Practice and Glycemic Control) After Controlling Covariates

**Figure 1:**
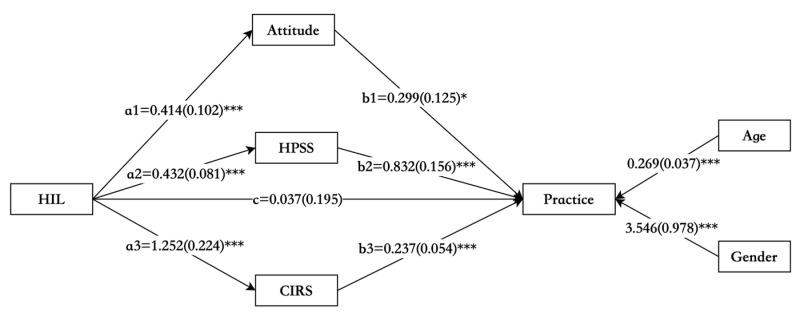
The parallel mediation model of HIL on self-management practice through self-management attitude, health problem-solving, and chronic disease resources (*n* = 225). Values are unstandardized estimate coefficients, and standard errors are shown in parentheses [51]. * *p* < 0.05, *** *p* < 0.001. HIL, health information literacy; HPSS, Health Problem-Solving Scale-Chinese version; CIRS, the Chronic Illness Resources Survey.

A parallel mediation analysis was conducted to explore the effect of associated factors on the relationship between HIL and health outcomes. In the analysis, self-management attitude, health problem-solving, and chronic disease resources were set as mediators, while self-management practice was the dependent variable, and HIL was considered as the independent variable. Age and gender were included as covariates in the model. As a result, health information literacy was significantly and positively linked to self-management attitude, health problem-solving, and chronic disease resources (a ranged from 0.414 to 1.252, with all *p* < 0.001); meanwhile, all three variables were significantly and positively correlated to self-management practice (b ranged from 0.237 to 0.832, with all *p* < 0.05). The total effect of the HILSS score on self-management practice was positive and significant (total effect = 0.817, 95% CI: 0.438 to 1.182). In addition, the indirect effect of the health information literacy on self-management practice through self-management attitude, health problem-solving, and chronic disease resources were all significant (total indirect effect = 0.780, 95% CI: 0.509 to 1.096). There was no direct effect between HIL and self-management practice (direct effect = 0.037, 95% CI: −0.338 to 0.427). The summary of parallel mediation analysis is displayed in Table 5 and Figure 1.

**Figure 2:**
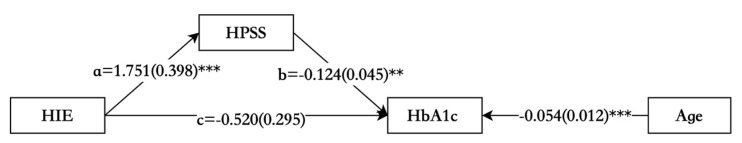
The mediation model of HIE on HbA1c through health problem-solving (*n* = 225). Values are unstandardized estimate coefficients, and standard errors are shown in parentheses [51]. ** *p* < 0.01, *** *p* < 0.001. HIE, health information evaluation; HPSS, Health Problem-Solving Scale-Chinese version; HbA1c, glycated hemoglobin.

Regarding glycemic control, we examined whether health problem-solving mediated the effect of health information evaluation (the only potential correlate) on HbA1c; age was included as covariate in the model. The result showed a significant indirect correlation between the health information evaluation and HbA1c through health problem-solving (a = 1.751, *p* < 0.001; b = −0.124, *p* < 0.01); however, the health information evaluation was not associated with HbA1c (c =−0.520, *p* = 0.078), which suggests complete mediation via health problem-solving. These findings indicated that participants with higher health information evaluation levels may tend to be more effective in health problem-solving, thereby achieving better glycemic control. The total and indirect effect of the health information evaluation on HbA1c were both significant (total effect = −0.737, 95% CI: −1.235 to −0.183; indirect effect = −0.217, 95% CI: −0.448 to −0.057). The summary of mediation analysis is displayed in Table 6 and Figure 2.

## 4. Discussion

Based on the SCT and empirical evidence, in the current study we examined whether HIL and related personal, behavioral, and environmental factors were potentially correlated to self-management practice and glycemic control in a sample of individuals with T2DM and MetS. We also explored how HIL affected health outcomes in this sample. The results were consistent with the theoretical framework and our hypotheses; they showed that self-management attitude, health problem-solving, and chronic disease resources were significantly correlated with self-management practice in managing MetS, and that they were significant mediators between HIL and self-management behaviors. Meanwhile, health information evaluation and health problem-solving were significantly correlated to HbA1c in this sample, and health problem-solving was a significant mediator between health information evaluation and glycemic control. These results illustrate the important role of HIL in the improvement of health outcomes in individuals with T2DM and MetS, especially the literacy related to health information evaluation. In this study, participants with higher HIL tend to have a solid self-care attitude, apply more effective health problem-solving skills, and access more resources and societal supports while managing their condition, thereby achieving better self-management practice and glycemic control.

In the current study, the level of HIL, self-management practice and glycemic control were suboptimal. Considering the score at ≥17.4 on the HILSS as the cutoff point for competent HIL suggested by previous research [29], more than half (53.6%) of the respondents failed to reach this threshold. However, the level of HIL was still slightly better than a sample of individuals with T2DM in a previous study [45], probably due to a lower age and higher education level of participants in the current sample, as age and education level are important influential factors of HIL [7]. With respect to related health outcomes, 37.3% of participants did not reach the 60% percentile over the total score on self-management practice, while a large proportion of the participants (78%) failed to achieve optimal glycemic control (HbA1c < 7.0%) in this sample. Adequate glycemic control is the key to prevent diabetes complications and serious cardiovascular diseases, with HbA1c < 7.0% (53 mmol/mol) being the recommended target level suggested by clinical guidelines [42]. These results were consistent with findings from previous research in which suboptimal self-management and glycemic control were reported among those with T2DM and those with MetS [35], highlighting the necessity of improving the HIL, glycemic control, and self-care practice regarding management of MetS. Coexistence of T2DM and MetS greatly increased the risk for developing more advanced cardiometabolic disease [52,53], such as stroke and myocardial infarction. This suggests that comprehensive management of diabetes along with the other indicators under MetS (lipid profile, blood pressure control, etc.) are particularly important to achieve optimal health outcomes in this population. In addition, the results of the current study are potentially relevant to global health issues, among which inadequate health information literacy, insufficient self-management, and poor glycemic control are commonly identified challenges among people with T2DM and MetS worldwide [52,54]. This study provided insight to further explore the potential role of health information literacy in various cultural contexts and, therefore, to develop tailored interventions addressing both indicators of T2DM and MetS simultaneously to improve health outcomes.

Identifying the influential factors associated with selected health outcomes is essential to understand their relationships. As indicated by the SCT, personal, behavioral, and environmental factors operate interactively and collectively impact human behavior change [38]. Based on the SCT, the findings of the current study supported the triadic relationship identified in the theoretical framework and partially supported our hypotheses. In the current sample, self-management attitude, health problem-solving, and chronic disease resources were significantly and positively related to self-management practice, and HIE and health problem-solving were significantly correlated to HbA1c. Congruent with previous research, self-management attitude refers to the awareness of the importance of various healthy behaviors, which is a significant predisposing factor influencing health behaviors among individuals with diabetes [14]. Previous reviews also demonstrated desirable effect of problem-solving on the self-management and control among individuals with diabetes [34,55]. Social support from family and friends, an essential component under the chronic disease resources, has been identified as an associated factor of diabetes self-management [56]. Previous studies have shown that HIL plays a moderating role in the relationship between knowledge and self-management behaviors in individuals with T2DM [57], but in the current study, there were no significant relationships identified between self-management knowledge and either practice or HbA1c. This discrepancy may suggest that self-management knowledge alone could not drive behavior change or better glycemic control, particularly in multi-morbidity, whereas practical skills such as health problem-solving may contribute more directly to influencing self-management and HbA1c in this population. Although interventions targeting the functional domain of the HL have shown significance in promoting better glycemic control, there was still limited evidence of the impact of strategies related to interactive or critical health literacy on HbA1c [20]. These results suggest that higher HIE, a subdomain of the critical HIL, is significantly associated with better glycemic control. Unlike information acquisition or application, which focus on access and basic utilization of information, HIE involves higher-order cognitive skills such as critical appraisal, judgment, and decision-making. These skills are particularly important for identifying related information and applying them in daily diabetes management. The ability to evaluate the credibility of online health information resources can directly influence a patient’s decision-making, thereby impacting glycemic control more intensively. On the other hand, subdomains such as information access may have a more indirect effect on clinical outcomes, as they do not necessarily guarantee the appropriate use of information. This finding underscores the importance of incorporating HIE training into diabetes education programs to enhance patients’ ability to make informed decisions and achieve better health outcomes.

Understanding the underlying mechanism in improving health outcomes is pivotal in identifying key concepts and developing tailored interventions. In the current study, the mediation analysis based on the SCT indicated that the overall HIL indirectly influenced self-management practice through attitude, health problem-solving, and chronic disease resources. Meanwhile, health problem-solving played a complete mediating role between HIE and HbA1c. These results implied that participants with higher HIL were likely to have a more decisive attitude, more effective health problem-solving, and utilize more available resources for effective self-management practice. As for glycemic control, participants with greater HIL and health problem-solving, especially health information evaluation, are more likely to have lower HbA1c. These findings are basically consistent with the conceptual framework and evidence from previous studies, in which researchers have identified that HIL influences some health promotion or health education constructs then affected health outcomes [33,58]. Moreover, the mediating effect of health belief between HIL and healthy lifestyles was also reported in both healthy adults and individuals with chronic disease [29,59]. However, in the previous research, there was limited evidence that HIL was an essential antecedent of health problem-solving, although problem-solving was mentioned in the conceptual model of health literacy pathways [21,59]. To the best of our knowledge, this study is one of the first to show indirect and direct pathways from HIL to health outcomes via health problem-solving among Chinese individuals with T2DM and MetS. These results provide evidence to support the application of problem-solving strategies in promoting HIL.

In addition, these findings showed that chronic illness resources play an essential role in mediating the impact of HIL on self-management practices, which is consistent with the theoretical framework and findings of previous research [59,60]. Health supporting environments, such as fitness parks and trails available in the community, have been found to be positively associated with better self-rated health status among community residents [31]. Meanwhile, utilization of informational support has demonstrated positive impact on health-promoting behaviors among older adults in Korea [30]. These results complemented the above evidence and showed that participants with higher HIL were more likely to access health supportive information and services. Therefore, providing resources may mitigate the adverse effect of low HIL on individuals with T2DM and MetS [6].

There are some implications for practice and future research based on the findings of the current study. These findings emphasized the urgent need to improve HIL, self-management practice, and glycemic control in individuals with T2DM and MetS. By exploring the pathways linking HIL and health outcomes, we identified several important mediators, including self-management attitudes, health problem-solving, and chronic illness resources. These findings highlight the necessities to enhance critical health literacy, which, as suggested by previous scholars, consists of two core components: reflection and action [61]. Digital health interventions have also been shown to effectively improve health information literacy, therefore providing scalable solutions for health education and self-management [18]. Furthermore, based on Hill-Briggs’ problem-solving model for chronic disease self-management, health problem-solving integrates disease-specific knowledge, transfer of past experience, problem-solving skills, and orientation [34]. Therefore, incorporation of such strategies, such as reflective practices [61], case-based learning, educational programs, or coaching sessions, as suggested by previous researchers [18,22], may be beneficial to enhance individuals’ critical HIL and problem-solving skills. These strategies aim to empower individuals to better navigate health information, solve health-related problems, and ultimately improve health outcomes. In addition, previous studies have shown that the effectiveness of health literacy interventions were limited, and they appeared to be heterogeneous among populations experiencing socioeconomic disadvantages [62]. Lower socioeconomic status is a key determinant of health in both the social gradient in health literacy and health disparities [63]. These findings highlight the importance of addressing modifiable factors, such as health information literacy and problem-solving skills, which could serve as crucial elements in tailoring effective strategies to ensure more equitable access to healthcare resources, including accessible and culturally sensitive health literacy interventions, community-based and educational programs, digital health solutions, and so on [58,64]. Through review of the literature, there is limited evidence examining the effect on self-management via interventions to enhance critical health information literacy. Future research should focus on developing and evaluating interventions to empower individuals with the skills needed to critically appraise and utilize health information. Additionally, longitudinal studies are needed to assess the sustained impact of HIL and associated factors on self-management outcomes over time. Expanding the demographic scope to include broader population variations would also provide a more comprehensive understanding of the role of HIL across diverse contexts.

There were some limitations in our study. First, the generalizability of findings may be limited as participants were enrolled from a single clinical site with a considerably small sample size. Moreover, the cross-sectional study design also prevents inferences of causality between study variables and health outcomes, although we employed path analysis. Therefore, a longitudinal study with a larger sample size is warranted to verify the relationships among study variables and health outcomes. Second, most of our data were self-reported, and this might have introduced information bias and misinterpretation of items during data collection. To reduce these biases, thorough explanation of the study procedure and sufficient time were ensured for consistency when collecting data from each participant. In addition, to more accurately capture the impact of diet, physical activity, and treatment adherence more accurately on HbA1c, future studies could employ objective measures, such as calculating caloric intake, using pedometers or fitness trackers to monitor physical activity, and leveraging electronic health records to assess treatment adherence. These approaches would help minimize measurement bias and provide a more precise understanding of the relationships among these variables. Third, although all instruments used in the current study demonstrated acceptable overall reliability, the Cronbach’s alpha for the HILSS health information evaluation subscale was slightly lower (0.55) than other subscales. This suggests that the items in this subdomain may not be measuring the construct as consistently as desired. Previous research reported that items of health information evaluation are often performance-based and tend to show lower internal consistency than perceived items [65]. In the future, researchers should focus on further refining these items in this subdomain to improve its reliability in order to better capture the health information evaluation concept. Through review of the literature, there is limited evidence examining the effect on self-management via interventions to enhance critical health information literacy. Future research should focus on developing and evaluating interventions to empower individuals with the skills needed to critically appraise and utilize health information. Additionally, longitudinal studies are needed to assess the sustained impact of HIL and associated factors on self-management outcomes over time. Expanding the demographic scope to include broader population variations would also provide a more comprehensive understanding of the role of HIL across diverse contexts.

## 5. Conclusions

In this study, a sample of individuals with T2DM and MetS showed insufficient HIL, inadequate self-management behavior, and suboptimal glycemic control, showing poor self-management of their condition. The results partially supported the hypotheses underlined by the Social Cognitive Theory in exploring the relationships between HIL and health outcomes. Based on the findings, we concluded that HIL has significant indirect and direct effects on self-management practice and HbA1c through self-management attitude, health problem-solving, and chronic illness resources. Therefore, healthcare providers need to enhance HIL, especially chronic illness resources literacy on health information evaluation, and target these modifiable factors in developing tailored interventions to improve health outcomes among individuals with T2DM and MetS.

## Figures and Tables

**Table 1:** Characteristics of the sample and Study Variables (*n* = 225).

Variables	N (%)	Mean ± SD or M(IQR)
Age, y		47.00 (18.00)
Gender		
Male	137(60.9)	
Female	88 (39.1)	
Residence		
Urban	166 (73.8)	
Rural	59 (26.2)	
Years of Education		12.00 (7.00)
Educational attainment		
Primary school	8 (3.6)	
Junior high	50 (22.2)	
High school	47 (20.9)	
College level	111 (49.3)	
Graduate level	9 (4.0)	
Employment status		
Full-time	123 (54.7)	
Retired	68 (30.2)	
Other	34 (15.1)	
Occupation		
Office clerks	67 (29.8)	
Workers	49 (21.8)	
Commercial personnel	45 (20.0)	
Service employees	49 (21.8)	
Farmers	15 (6.7)	
Household income(monthly), CNY		
<3000	13 (5.8)	
3000–4999	40 (17.8)	
5000–7999	44 (19.6)	
≥8000	128 (56.9)	
Marital status		
Married	179 (79.6)	
Unmarried	46 (20.4)	
Medical costs		
Medical insurance	215 (95.6)	
Other	10 (4.4)	
HILSS		16.83 ± 2.96
HIC		3.00 (0.75)
HIS		6.19 ± 1.53
HIE		3.20 (0.85)
HIA		2.32 (0.75)
HIM		2.50 (0.75)
Knowledge		19.00 (4.00)
Attitude		37.00 (6.00)
HPSS		17.22 (4.81)
CIRS		62.12 ± 11.74
Practice		42.00 (15.00)
Body mass index, kg/m^2^		27.37 (5.20)
≥24	189 (84.0)	
Waist, cm		97.00 (12.50)
Comorbidities, n		
Hypertension	110 (48.9)	
Dyslipidemia	194 (86.2)	
Coronary heart disease	27 (12.0)	
Ischemic stroke	20 (8.9)	
Systolic Blood pressure		133.00 (21.00)
Diastolic Blood pressure		84.00 (13.00)
Triglyceride, mmol/L		1.96 (1.63)
Total cholesterol, mmol/L		4.99 (1.66)
Lipoprotein, mmol/L		1.12 (0.41)
HDL		3.22 ± 1.00
LDL		16.83 ± 2.96
HbA1c, mmol/L		8.80 (3.55)
≥7.0 (%)	175 (77.8)	

Abbreviations: SD, standard deviation; M, median; IQR, inter-quartile range; y, year; CHY, Chinese Yuan; cm, centimeter; kg, kilogram; m, meter; HDL, high-density lipoprotein cholesterol; LDL, low-density lipoprotein cholesterol; HILSS, Health Information Literacy Self-rating Scale; HIC, health information consciousness; HIS, health information access; HIE, health information evaluation; HIA, health information application; HIM, health information morality; HPSS, Health Problem-Solving Scale-Chinese version; CIRS, the Chronic Illness Resources Survey; HbA1c, glycated hemoglobin.

**Table 2:** Bivariate correlation coefficients of the study variables (*n* = 225).

Variables	Age	YED	HILSS	Knowledge	Attitude	HPSS	CIRS	Practice	HbA1c
Age	1								
YED	−0.454 **	1							
HILSS	−0.190 **	0.399 **	1						
Knowledge	0.020	0.143 *	0.354 **	1					
Attitude	−0.041	0.117	0.308 **	0.431 **	1				
HPSS	0.094	0.023	0.379 **	0.302 **	0.434 **	1			
CIRS	0.140 *	0.072	0.353 **	0.362 **	0.499 **	0.454 **	1		
Practice	0.457 **	−0.211 **	0.207 **	0.321 **	0.392 **	0.519 **	0.516 **	1	
HbA1c	−0.306 **	0.148 *	−0.146 *	−0.178 **	−0.084	−0.289 **	−0.227 **	−0.351 **	1

Notes: ** *p* < 0.01, * *p* < 0.05; Abbreviations: YED, Years of Education; HILSS, Health Information Literacy Self-rating Scale; HPSS, Health Problem-Solving Scale-Chinese version; CIRS, the Chronic Illness Resources Survey; HbA1c, glycated hemoglobin.

**Table 3:** Hierarchical multiple regression for Self-Management Practice (*n* = 225).

	Model 1					Model 2				
Variables	B	SE	*t*	*p* Value	95% CI	B	SE	*t*	*p* Value	95% CI
Age	0.324	0.051	6.379	<0.001	0.224; 0.424	0.254	0.040	6.395	<0.001	0.176; 0.332
Gender	4.669	1.243	3.755	<0.001	2.218; 7.119	3.031	1.006	3.012	0.003	1.048; 5.014
YED	0.013	0.217	0.061	0.951	−0.414; 0.441	−0.186	0.167	−1.111	0.268	−0.516; 0.144
Employment status	−0.975	0.876	−1.113	0.267	−2.701; 0.751	−0.761	0.669	−1.137	0.257	−2.079; 0.558
Household income	−0.153	0.723	−0.212	0.833	−1.578; 1.272	−0.651	0.560	−1.161	0.247	−1.755; 0.454
Attitude						0.330	0.126	2.621	0.009	0.082; 0.578
HPSS						0.853	0.146	5.852	<0.001	0.566; 1.140
CIRS						0.238	0.050	4.805	<0.001	0.141; 0.336

Model 1 statistics: R^2^ = 0.249, adjusted R^2^ = 0.232, F = 14.501, *p* < 0.001; Model 2 statistics: R^2^ = 0.568, adjusted R^2^ = 0.552, F = 6.869, *p* < 0.01. Abbreviations: B, unstandardized coefficient; SE, standard error, CI, confidence interval; YED, Years of Education; Attitude, Self-management attitude subscale; HPSS, Health Problem-Solving Scale-Chinese version; CIRS, the Chronic Illness Resources Survey.

**Table 4:** Hierarchical multiple regression for HbA1c (*n* = 225).

	Model 1					Model 2				
Variables	B	SE	*t*	*p* Value	95% CI	B	SE	*t*	*p* Value	95% CI
Age	−0.054	0.013	−4.07	<0.001	−0.080; −0.028	−0.050	0.013	−3.896	<0.001	−0.076; −0.025
YED	0.018	0.051	0.350	0.727	−0.083; 0.119	0.072	0.053	1.377	0.170	−0.031; 0.176
Employment status	0.133	0.226	0.589	0.557	−0.312; 0.578	0.126	0.218	0.58	0.563	−0.303; 0.555
HPSS						−0.119	0.044	−2.683	0.008	−0.207; −0.032
HIE						−0.643	0.263	−2.446	0.015	−1.161; −0.125

Model 1 statistics: R^2^ = 0.086, adjusted R^2^ = 0.074, F = 6.969, *p* < 0.001; Model 2 statistics: R^2^ = 0.163, adjusted R^2^ = 0.144, F = 5.466, *p* < 0.05. Abbreviations: B, unstandardized coefficient; SE, standard error, CI, confidence interval; YED, Years of Education; HPSS, Health Problem-Solving Scale-Chinese version; HIE, health information evaluation.

**Table 5:** Summary of Indirect Effect for the Parallel Mediation Model (*n* = 225).

				95% CI	
Paths	Effect	SE	*p* Value	Lower	Upper
Path1: HIL to Attitude to Practice	0.124	0.062	0.044	0.026	0.269
Path2: HIL to HPSS to Practice	0.360	0.104	0.001	0.177	0.583
Path3: HIL to CIRS to Practice	0.297	0.083	<0.001	0.161	0.494
Total indirect effect	0.780	0.152	<0.001	0.509	1.096
Direct effect: HIL to Practice	0.037	0.195	0.850	−0.338	0.427
Total effect: HIL to Practice	0.817	0.189	<0.001	0.438	1.182

**Table 6:** Summary of mediating effects of HPSS between HIE and HbA1c (*n* = 225).

				95% CI	
Paths	Effect	SE	*p* Value	Lower	Upper
Total indirect effect:	−0.217	0.101	0.032	−0.448	−0.057
Direct effect:	−0.520	0.295	0.078	−1.094	0.074
Total effect:	−0.737	0.265	0.005	−1.235	−0.183

## Data Availability

Data will be available from the corresponding author upon request.

## Supplementary Materials

The following supporting information can be downloaded at: https://www.mdpi.com/article/10.3390/nursrep15030090/s1, Figure S1: Flow diagram for participants recruitment; Table S1: Reporting Checklist; Table S2: Cronbach’s alpha of Study Variables; Table S3: Sociodemographic differences on Self-management Practice and HbA1c.

## Public Involvement Statement

Eligible patients were involved. They completed a self-reported questionnaire.

## Guidelines and Standards Statement

This manuscript was drafted against the guidelines of the Strengthening the Reporting of Observational Studies in Epidemiology (STROBE) for cross-sectional studies via the equator network: https://www.equator-network.org/.
