# Supplementary material for: Association of Health Information Literacy and Health Outcomes Among Individuals with Type 2 Diabetes and Metabolic Syndrome
Source: Nurs Rep. 2025 Mar 5;15(3):90. doi: 10.3390/nursrep15030090 (PMC11944319; doi:10.3390/nursrep15030090)
Supplement: Supplementary file 1 [file nursrep-15-00090-s001.zip › Supplementary Table S2-3.pdf]

Supplementary Table S2. Cronbach's alpha of Study Variables (n = 225)

		Cronbach's alpha		
Variable	items	Original study	Chinese population	Current study
HILSS	29	0.847	0.903	0.844
HIE	5	0.832	0.832	0.559
SMKAP-Mets	46	0.836	0.836	0.844
Knowledge	24	0.873	0.873	0.706
Attitude	9	0.792	0.792	0.743
Practice	13	0.793	0.793	0.815
HPSS	30	0.890	0.911	0.746
CIRS	19	0.820	0.845	0.802

Note. HILSS, Health Information Literacy Self-Rating Scale; HIE, health information evaluation; SMKAP-Mets, Self-management knowledge, attitude and practice for metabolic syndrome; HPSS, Health Problem-Solving Scale; CIRS, the Chronic Illness Resources Survey.

Supplementary Table S3. Sociodemographic differences on Self-management Practice and HbA1c (n = 225)

Variable	Self-management Practice			HbA1c		
	Mean±SD/ M(IQR)	Statistic	*p*	Mean±SD/ M(IQR)	Statistic	*p*
Age						
<45	37.31±7.51	*H*=54.496	<0.001***	9.70±2.36	*H*=20.961	<0.001***
45-59	44.59±9.29			8.82±2.17		
≥60	49.18±8.67			7.40(2.80)		
Gender						
Male	39.00(13.00)	*Z*=-4.725	<0.001***	9.11±2.33	*Z*=-1.107	0.268
Female	46.25±8.92			8.45(3.47)		
Residence						
Urban	43.10±9.74	*Z*=-1.172	0.241	8.65(3.60)	*Z*=-0.887	0.375
Rural	40.00(16.00)			9.13±2.16		
Educational attainment						
Primary school	44.75±9.54	*H*=7.617	0.107	8.53±2.23	*H*=5.105	0.277
Junior high	45.66±9.82			8.38±2.04		
High school	42.13±8.85			9.21±2.49		
College level	41.53±9.82			9.10(3.60)		
Graduate level	39.00(6.00)			9.96±2.85		
Employment status						
Full-time	39.00(11.00)	*H*=35.235	<0.001***	9.30(3.90)	*H*=11.887	0.003**
Retired	48.44±8.85			7.55(3.10)		
Other	40.09±9.30			9.31±1.96		
Occupation						
Office clerks	44.21±10.75	*H*=5.180	0.269	8.10(3.80)	*H*=3.265	0.514
Workers	40.31±9.04			8.89±2.34		
Commercial personnel	41.71±9.01			9.45±2.40		
Service industry	43.57±9.45			9.01±2.19		
Farmers	43.07±8.60			8.75±1.81		
Household income(monthly), CNY						
<3000	48.62±7.50	*H*=9.244	0.026*	8.70±1.80	*H*=2.046	0.563
3000-4999	44.48±10.77			2.73±2.32		
5000-7999	41.50(14.50)			8.10(4.30)		
≥8000	40.50(14.00)			9.16±2.36		
Marital status						
Married	41.00(14.00)	*Z*=-1.042	0.298	8.70(3.50)	*Z*=-0.837	0.403
Other	44.04±9.31			9.20±2.32		
Medical costs						
Medical insurance	41.00(14.00)	*Z*=-0.860	0.390	8.80(3.60)	*Z*=-0.442	0.658
Other	40.4±8.30			9.24±2.26		

Abbreviations: SD, standard deviation; M, median; IQR, inter-quartile range; y, year; CHY, Chinese Yuan; HbA1c, glycated haemoglobin.

Z for Mann-Whitney *U*-test; *H* for Kruskal-Wallis test.

*** $p < 0.001$, ** $p < 0.01$, * $p < 0.05$
