# Supplementary figures and images for: Association of Health Information Literacy and Health Outcomes Among Individuals with Type 2 Diabetes and Metabolic Syndrome
Source: Nurs Rep. 2025 Mar 5;15(3):90. doi: 10.3390/nursrep15030090 (PMC11944319; doi:10.3390/nursrep15030090)

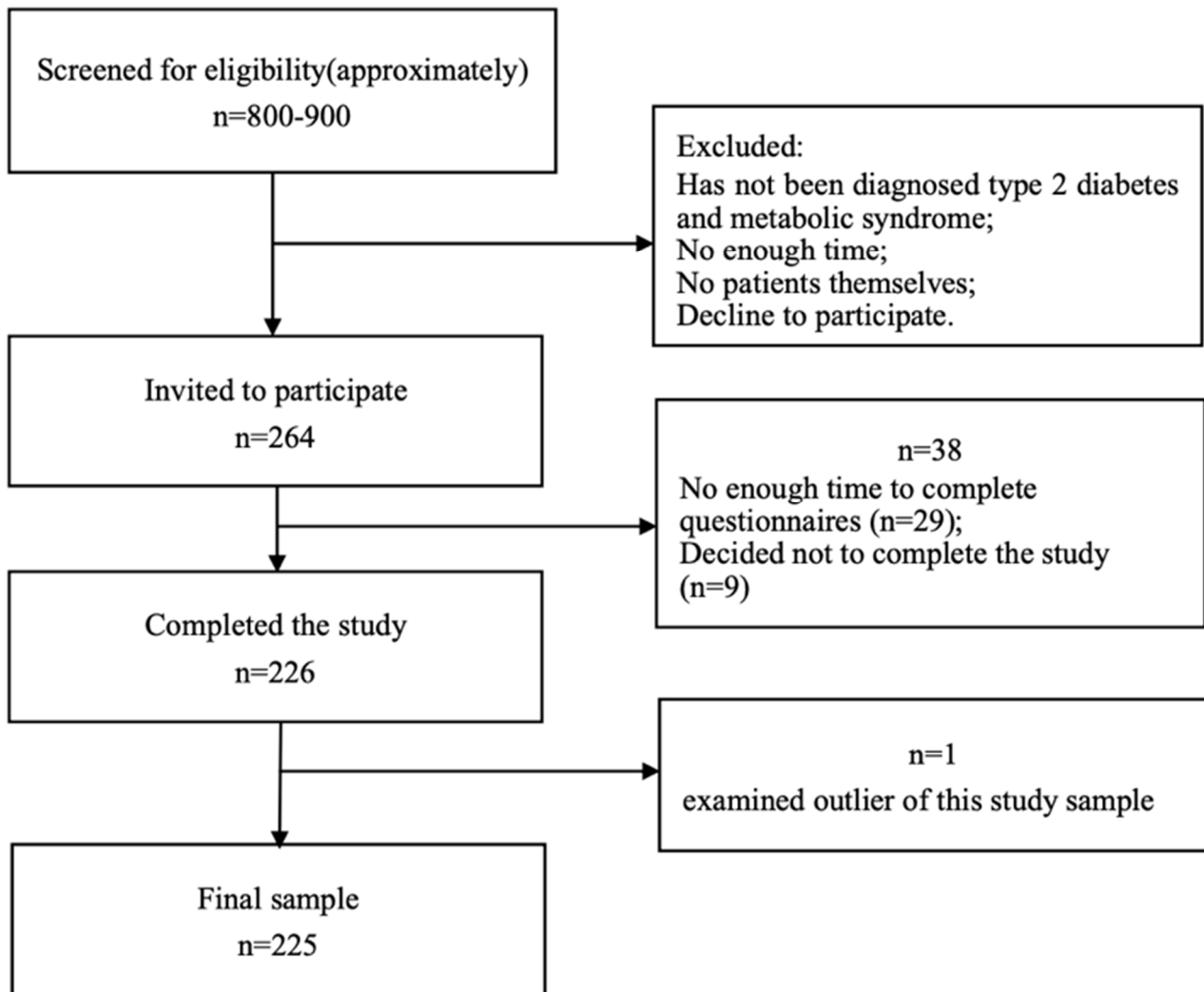

Figure S1. Flow diagram for participants recruitment

Supplement: Supplementary file 1 [file nursrep-15-00090-s001.zip › Supplementary Figure S1 Flow diagram.pdf]
